# Supplementary material for: Direct Photochemical Synthesis of Substituted Benzo[b]fluorenes
Source: Org Lett. 2024 Nov 25;26(48):10364–8. doi: 10.1021/acs.orglett.4c03978 (PMC11629382; doi:10.1021/acs.orglett.4c03978)
Supplement: Supplementary file 2 — ol4c03978_si_002.zip [file ol4c03978_si_002.zip › FID for Publication/5d/20240424_RC-2-C-5-69_01/plots/RC-2-C-5-69_HSQCAD_20240424_01_plot01.pdf]

RC-2-C-5-69

Location **3**	Pulse sequence **HSQCAD**	Temperature **25**	Suboperator **RuairiCrawford**
Date collected **2024-04-24**	Solvent **dmso**	Spectrometer **Agilent-NMR-vnmrs500**	Operator **MBaumann**

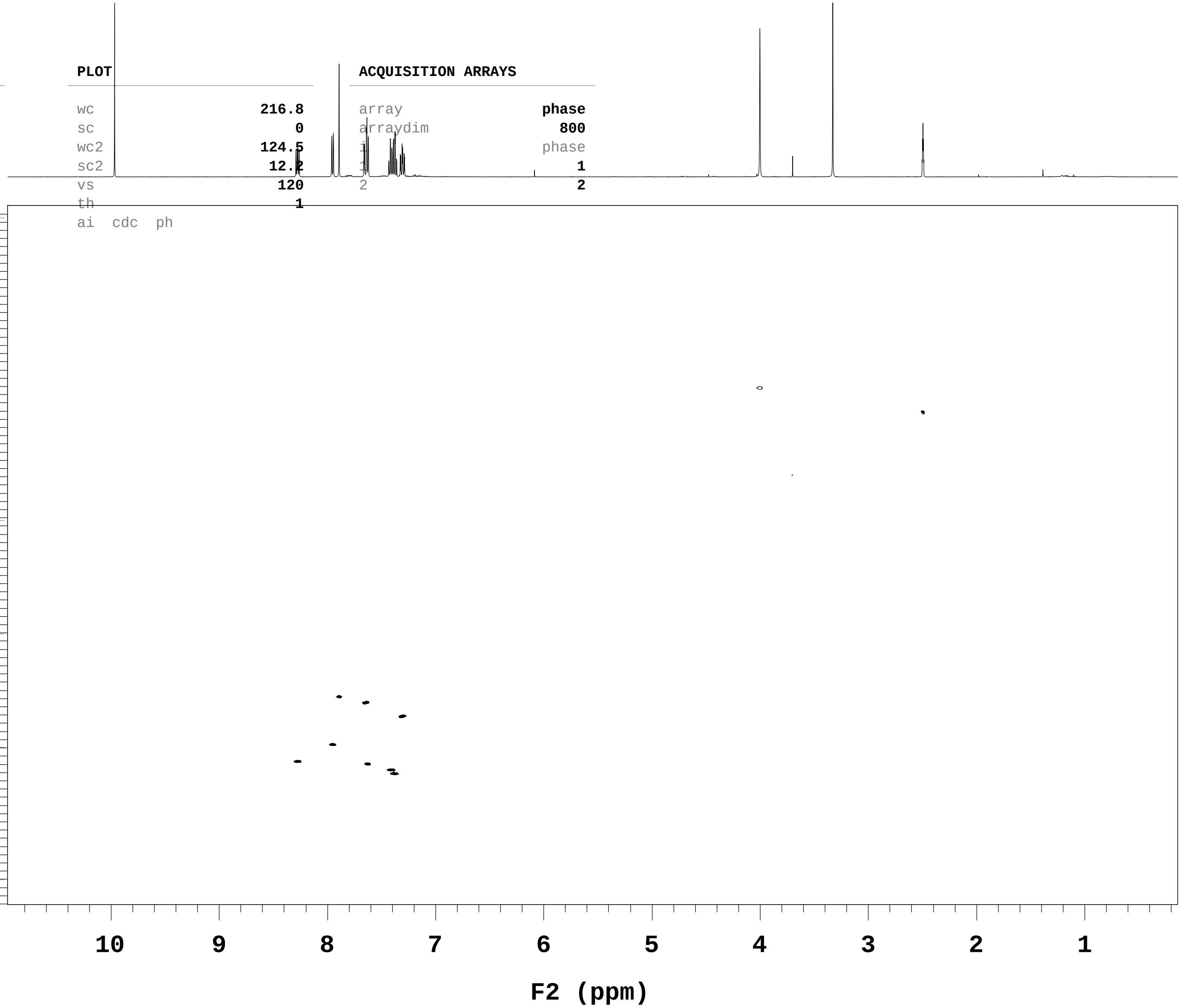

SAMPLE

date	Apr 24 2024
solvent	dmso
sample	20240424_RC-2-C-5-69_01

ACQUISITION

sw	5411.3
at	0.150
np	1624
fb	4000
ss	32
d1	1.000
nt	4

2D ACQUISITION

sw1	21373.2
ni	400
phase	arrayed

PRESATURATION

satmode	n
wet	n

TRANSMITTER

tn	H1
sfrq	500.035
tof	271.0
tpwr	62
pw	7.200

DECOUPLER

dn	C13
dof	-2486.7
dm	nny
decwave	W40_One_NMR_new
dmf	32258
dpwr	39
pwxlvl	59
pwx	10.000

HSQC

j1xh	146.0
nullflg	y
mult	2

FLAGS

hs	nn
sspul	y
PFGflg	y
hsglvl	6348

ADIABATIC

pwx180ad	One_NMR_new_ad300
pwx180adR	One_NMR_new_ad300R
pwx180	465.4
pwxlvl180	53
pwx180ref	One_NMR_new_ref200
pwx180r	2000.2
pwxlvl180r	45

SPECIAL

temp	25.0
gain	40
spin	0

F2 PROCESSING

gf	0.069
gfs	not used
fn	2048

F1 PROCESSING

gf1	0.017
gfs1	not used
proc1	lp
fn1	4096

DISPLAY

sp	[illegible]
wp	5411.3
sp1	-1257.2
wp1	21373.2
rfl	-65.5
rfp	0
rfl1	1257.2
rfp1	0

PLOT

wc	216.8
sc	0
wc2	124.5
sc2	12.2
vs	120
th	1
ai cdc ph	

ACQUISITION ARRAYS

array	phase
arraydim	800
1	phase
1	1
2	2

Plotname: RC-2-C-5-69_HSQCAD_20240424_01_plot01

Data file /home/data/MBaumann/newucd500/RuairiCrawford/2024/20240424_RC-2-C-5-69_01/RC-2-C-5-69_HSQCAD_20240424_01.fid

Plot date 2024-04-24
